# The role of adjuvant pelvic radiotherapy in rectal cancer with synchronous liver metastasis: a retrospective study

**DOI:** 10.1186/1748-717X-5-75

**Published:** 2010-08-31

**Authors:** Jun Won Kim, Yong Bae Kim, Nam-Kyu Kim, Byung-Soh Min, Sang Joon Shin, Joong Bae Ahn, Woong Sub Koom, Jinsil Seong, Ki Chang Keum

**Affiliations:** 1 Department of Radiation Oncology, Yonsei Cancer Center, Yonsei University College of Medicine, Yonsei University Health System, 134 Sinchon-dong, Seodaemun-gu, Seoul, 120-752, Korea; 2 Department of Surgery, Yonsei Cancer Center, Yonsei University College of Medicine, Yonsei University Health System, 134 Sinchon-dong, Seodaemun-gu, Seoul, 120-752, Korea; 3 Department of Internal Medicine, Yonsei Cancer Center, Yonsei University College of Medicine, Yonsei University Health System, 134 Sinchon-dong, Seodaemun-gu, Seoul, 120-752, Korea

## Abstract

**Background:**

Synchronous liver metastases are detected in approximately 25% of colorectal cancer patients at diagnosis. The rates of local failure and distant metastasis are substantial in these patients, even after undergoing aggressive treatments including resection of primary and metastatic liver tumors. The purpose of this study was to determine whether adjuvant pelvic radiotherapy is beneficial for pelvic control and overall survival in rectal cancer patients with synchronous liver metastasis after primary tumor resection.

**Methods:**

Among rectal cancer patients who received total mesorectal excision (TME) between 1997 and 2006 at Yonsei University Health System, eighty-nine patients diagnosed with synchronous liver metastasis were reviewed. Twenty-seven patients received adjuvant pelvic RT (group S + R), and sixty-two patients were managed without RT (group S). Thirty-six patients (58%) in group S and twenty patients (74%) in group S+R received local treatment for liver metastasis. Failure patterns and survival outcomes were analyzed.

**Results:**

Pelvic failure was observed in twenty-five patients; twenty-one patients in group S (34%), and four patients in group S+R (15%) (*p *= 0.066). The two-year pelvic failure-free survival rates (PFFS) of group S and group S+R were 64.8% and 80.8% (*p *= 0.028), respectively, and the two-year overall survival rates (OS) were 49.1% and 70.4% (*p *= 0.116), respectively. In a subgroup analysis of fifty-six patients who received local treatment for liver metastasis, the two-year PFFS were 64.9% and 82.9% (*p *= 0.05), respectively; the two-year OS were 74.1% and 80.0% (*p *= 0.616) in group S (n = 36) and group S+R (n = 20), respectively.

**Conclusions:**

Adjuvant pelvic RT significantly reduced the pelvic failure rate but its influence on overall survival was unclear. Rectal cancer patients with synchronous liver metastasis may benefit from adjuvant pelvic RT through an increased pelvic control rate and improved quality of life.

## Background

According to the data on cancer incidence between 2003 and 2005 from the Korea Central Cancer Registry, colorectal cancer (CRC) is the fourth most common cancer in men (37.9%) after cancers of the stomach (66.0%), lung (48.5%), and liver (44.9%). According to the same data set, colorectal cancer is the fourth most common cancer in Korean women (28.0%) after breast (37.3%), thyroid (36.2%), and stomach (34.1%) cancers. When the annual incidence of CRCs in 2005 was compared to that in 1999, there was an increase of 150% in men and 135% in women; CRC was shown to be one of the most sharply increased malignancies in Korea [1]. The annual disease-specific death rate for colorectal cancer is approximately 40% and liver metastases are found in approximately two-thirds of these patients [2], while synchronous liver metastases are found in 20% to 30% of colorectal cancer patients at initial diagnosis [3].

In rectal cancer patients with liver metastasis, conservative management including diverting colostomy resulted in a median survival of approximately three to five months, while resection of the primary tumor increased median survival to fourteen to twenty-four months [4-6]. Resection of both the primary and metastatic liver tumors resulted in a median survival of thirty-seven months and a five-year survival rate of 25-35% [7,8]. Despite an increased chance of survival following resection of the primary and metastatic liver tumors, the reported rate of pelvic failure was approximately 30-35% and the rate of extra-hepatic metastases was up to 67% [9,10]. The cause of death for many patients was either pelvic failure or distant extra-hepatic metastases. The rate of treatment failure is currently on the rise because patients live longer due to improved efficacy of treatment modalities; pelvic failures or uncontrolled primary tumors may threaten patient survival and quality of life in these cases.

According to 2010 National Comprehensive Cancer Network (NCCN) guideline, preoperative CCRT has become the standard care in locally advanced rectal cancer. However, when patients are diagnosed with synchronous liver metastasis, preoperative CCRT is no longer a part of standard care, and many physicians decide against postoperative CCRT even at high risk of local recurrence. Despite the consensus that adjuvant pelvic RT provides survival benefit for stage II and III rectal cancer [11], the role of adjuvant pelvic RT in rectal cancer with synchronous liver metastasis has not been defined. In this study, we investigated the clinical implications of adjuvant pelvic RT following primary tumor resection in rectal cancer patients with synchronous liver metastasis.

## Methods

### Patient eligibility

Between 1997 and 2006, 306 patients with rectal cancer and synchronous liver metastasis were treated at Yonsei Cancer Center, Severance Hospital (Seoul, Republic of Korea). Synchronous liver metastasis was diagnosed during work-up, at the time of operation, or within three months after definitive treatment. A total of eighty-nine patients who underwent surgical resection of the primary tumor and/or local treatment for liver metastasis were retrospectively analyzed. Patients who did not receive resection of the primary tumor were excluded from this study. Patients who had synchronous extra-hepatic metastases, pathology other than adenocarcinoma, inadequate medical records, or patients who received preoperative chemoradiation or refused recommended treatments were also excluded from the study. Each patient was evaluated through history, physical examination, routine blood tests, chest radiography, and other relevant studies. Pretreatment studies included computerized tomography (CT) or magnetic resonance imaging (MRI) of the primary tumor site. Patients were routinely evaluated with abdominal-pelvic CT for liver metastasis.

**Figure 1:**
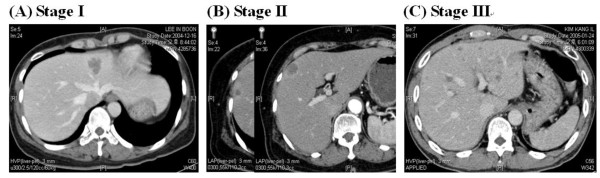
**Pettavel and Morgenthaler's Staging for colorectal hepatic metastases**. (A) CT imaging for stage I, (B) CT imaging for stage II, and (C) MRI imaging for stage III disease.

Since all patients had liver metastasis without presence of extrahepatic metastasis at diagnosis, we used Duke staging to describe the status of primary tumor and pelvic lymph node metastasis. The liver metastasis status was classified according to Pettavel and Morgenthaler staging [12]. Stage I was defined as a solitary or small metastasis, stage II was defined as two or three metastases with a maximum diameter less than or equal to 2 cm, and stage III was defined as numerous and large metastases or the presence of hepatomegaly and/or ascites (Table 1 and Figure 1). Among 89 patients reviewed, 27 patients received adjuvant pelvic RT (group S + R), and 62 patients were managed without adjuvant RT (group S). The median follow-up periods were twenty-five months (range 6 to 138 months) for all 89 patients and 62 months (range 22 to 138 months) for surviving patients (n = 28). There was no significant difference in the median follow up periods of 34 months (range 6-138 months) for group S+R and 24 months (rage 6-96 months) for group S.

**Table 1:** Pettavel and Morgenthaler's Staging for Colorectal Hepatic Metastases

Stage I	solitary or small
Stage II	few (maximum diameter = 2 cm)
Stage III	numerous and large (hepatomegaly, ascites)

### Treatment profiles

All patients included in this study received total mesorectal excision (TME) by means of either low anterior resection (n = 72), abdominoperineal resection (n = 13), or Hartmann's operation (n = 4). Treatments for metastatic liver tumors included lobectomy, wedge resection, radiofrequency ablation (RFA), and transarterial chemoembolization (TACE) with intra-arterial chemotherapy for localized liver metastases; systemic chemotherapy was administered for extensive liver metastases. Hepatic resection was performed in a patient whose metastatic hepatic tumor was determined to be resectable based on location of tumor, extent of disease, and adequate hepatic function. For metastatic tumors smaller than 3 cm in diameter, RFA was recommended as an alternative treatment for patients who were not candidates for surgery due to the poor anatomical location of the liver metastases, insufficiency in the functional hepatic reserve following a resection, or a co-morbidity that prohibited major surgery. All modalities except TACE plus intra-arterial chemotherapy were considered local treatments for liver metastasis.

Twenty-four out of 27 patients who received postoperative pelvic RT underwent adjuvant 5-FU/leucovorin (FL) chemotherapy. The regimen consisted of a 5-FU (450 mg/m^2^/day) intravenous bolus infusion with leucovorin (20 mg/m^2^/day) for five consecutive days every four weeks for twelve cycles. The first two cycles of FL chemotherapy were given alone following surgery, with the remainder administered concurrently with radiation. To three patients, who were treated between 1997 and 2000 but did not receive postoperative pelvic RT, adjuvant FL chemotherapy was given up to 12 cycles. Fifty-nine patients who were treated after the year 2000 and did not receive postoperative pelvic RT were subject to adjuvant chemotherapy with 5-FU/leucovorin/oxaliplatin (FOLFOX) or 5-FU/leucovorin/irrinotecan (FOLFIRI). The FOLFOX regimen consisted of oxaliplatin 85 mg/m^2 ^on day one, followed by a bolus of 5-FU at 400 mg/m^2 ^and LV at 200 mg/m^2 ^on days one and two with infusional 5-FU at 600 mg/m^2 ^for twenty-two hours on days one and two. The FOLFIRI regimen consisted of irinotecan at 180 mg/m^2 ^on day one with leucovorin at 400 mg/m^2 ^administered as a two hour infusion before 5-FU at 400 mg/m^2 ^being administered as an intravenous bolus injection, followed by 5-FU at 600 mg/m^2 ^as a twenty-two hour infusion immediately after the 5-FU bolus injection on days one and two.

The postoperative radiation treatment consisted of mega-voltage photon beams delivered either in a three-field plan (posteroanterior and two lateral fields) or a four-field box plan. The top of the treatment field was placed at the L4-5 junction, with the lateral borders 1-2 cm outside the bony pelvis, and the inferior margin at 4 cm below the tumor. A total dose of 41.4 to 45 Gy was delivered to the whole pelvis including the tumor bed and pelvic lymph nodes and a boost dose of 9 to 12.6 Gy was delivered to the tumor bed in a 1.8 Gy daily fraction. Boost volume consisted of areas of initial tumor, surrounding mesorectum, anastomosis site and presacral area.

Because patients were diagnosed with synchronous liver metastasis, the NCI guideline for postoperative pelvic RT was not strictly followed and decisions for adjuvant pelvic RT were made at physicians' discretion. In Table 2, although not statistically significant, group S+R shows higher rates of lymph node metastasis (Duke C), localized liver metastasis (liver stage I or II), and liver resection. These results indicate that patients with controlled liver metastasis who had a higher risk of pelvic recurrence were subjected to postoperative pelvic RT. At our institution, patients who underwent adjuvant chemotherapy with FOLFOX or FOLFIRI regimen were not recommended for concurrent radiotherapy due to increased toxicity and this result is shown in Table 2.

**Table 2:** Patient Characteristics (n = 89)

	S	S + RT	P value
No. of Patients	62	27	
Sex (M:F)	45:17	19:8	
Age (years)(mean)	25-80 (57)	23-66 (53)	
Duke stage			0.211
A or B	13 (21%)	3 (11%)	
C	49 (79%)	24 (89%)	
Liver mets stage			0.561
I or II	28 (45%)	14 (52%)	
III	34 (55%)	13 (48%)	
Pelvic surgery			
APR	9 (15%)	4 (15%)	
LAR	51 (82%)	21 (78%)	
Hartmann	2 (3%)	2 (7%)	
Lateral resection margin			NS
Positive	10 (16%)	3 (11%)	
Negative	52 (84%)	24 (89%)	
Liver surgery			0.151
Resection	22 (61%)	16 (80%)	
RFA	4 (11%)	2 (10%)	
Resection + RFA	10 (28%)	2 (10%)	
Chemotherapy			
FL	3	24	
FOLFOX	37	2	
FOLFIRI	22	1	

Liver metastasis staging according to Pettavel and Morgenthaler

*Abbreviations: *APR = abdominoperineal resection; LAR = low anterior resection; RFA = radiofrequency ablation; FL = 5-FU/leucovorin; FOLFOX = 5-FU/leucocovorin/oxaliplatin; FOLFIRI = 5-FU/leucovorin/irrinotecan.

### Statistical analysis

The primary endpoint of the study was pelvic failure free survival (PFFS). PFFS was defined as any relapse within the pelvic cavity. The PFFS rate and overall actuarial survival rate were calculated. Actuarial curves were plotted using the Kaplan-Meier method; tests of significance among actuarial data were based on the log-rank statistic. Multivariate proportional hazards regression analysis was done using standard techniques with the log-linear hazard function of the Cox model. The prognostic factors that revealed *p *values < 0.30 in univariate analysis were included for multivariate analysis. Differences between means, proportions, and distributions were evaluated by Chi-square testing. For actuarial data, *p *values ≤ 0.05 were considered statistically significant.

## Results

### Patient characteristics

The median age was 56 years (range 23-80 years), and 72% of the study population was male. Duke stage C was reported in 73 patients (82%), and 47 patients (53%) were diagnosed with liver metastasis stage III. LAR was the most frequently performed type of surgery for resection of the primary tumor (81%). For the 56 patients who received local treatment for liver metastasis, resection including and lobectomy and wedge resection was the most frequently used method (68%).

We divided the patient population into two groups; group S+R consisted of twenty-seven patients who received resection of the primary tumor and adjuvant pelvic RT and group S, which consisted of sixty-two patients who were managed with primary tumor resection without pelvic irradiation. Patient characteristics and clinical profiles are listed in Table 2. No significant differences were observed in the distribution of primary and metastatic liver stages or in the local treatment for liver metastasis between the two groups. Liver metastasis stage III was more frequently found in group S than in group S+R (55% vs. 48%) with no statistical significance. The number of patients who received local treatment for liver metastasis was thirty-six (58%) in group S and twenty (74%) in group S+R. Positive lateral margin from primary tumor resection was found in 10 patients (16%) from group S and 3 patients (11%) from group S+R with no significant difference between the two groups.

### Treatment outcome

**Figure 2:**
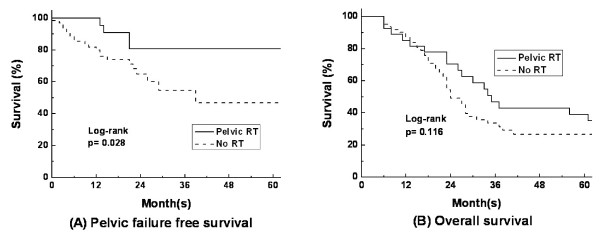
**Survival of rectal cancer patients with synchronous liver metastasis after postoperative pelvic RT**. (A) Pelvic failure-free survival and (B) Overall survival rates.

The median two-year overall survival for all 89 patients was 55.7%. The two-year overall survival was 49.1% for group S and 70.4% for group S+R, and no significant difference in overall survival was observed between the two groups (*p *= 0.116) (Figure 2B). No radiation induced gastrointestinal toxicity greater than Radiation Therapy Oncology Group (RTOG) grade 2 was noted in group S+R. During follow-up, pelvic failure was observed in twenty-five patients; twenty-one patients (34%) in group S and four patients (15%) in group S+R (*p *= 0.066). Forty-one patients developed distant metastases, and there was no significant difference between group S and group S+R in this regard (42% vs. 56%, *p *= 0.236, respectively). Patterns of failure are summarized in Table 3. The two-year pelvic failure-free survival rates of group S and group S+R were 64.8% and 80.8%, respectively. Group S+R demonstrated a significantly higher pelvic failure-free survival rate (*p *= 0.028) (Figure 2A). Among the four patients with pelvic failure in group S+R, one patient received salvage surgery and RT, two patients receive chemotherapy and one patient received palliative RT and chemotherapy. Among the twenty-one patients who experienced pelvic failure in group S, one patient received salvage surgery, four patients received palliative RT and chemotherapy, eight patients received chemotherapy and seven patients were managed with conservative care only.

**Table 3:** Patterns of Treatment Failure

	No. of Patients		
		
Failure pattern	S (n = 62)	S + RT (n = 27)	P value
Pelvic failure	21 (34%)	4 (15%)	0.066
Distant failure	26 (42%)	15 (56%)	0.236

A univariate analysis was carried out on clinical factors including patients' age, Duke stage, initial CEA level, lateral resection margin, liver metastasis stage, local treatment for liver metastasis, and adjuvant pelvic RT, in order to determine their influence on pelvic control and overall survival. For pelvic failure-free survival, positive lateral resection margin of the primary tumor and adjuvant pelvic RT were significantly related with improved pelvic failure-free survival, and their independent association with survival was verified by a multivariate analysis (Table 4). Local treatment for liver metastasis demonstrated a significant correlation with improved overall survival. Although not statistically significant, positive lateral resection margin of the primary tumor and initial CEA > 100 ng/ml demonstrated adverse correlation with regards to overall survival. In a multivariate analysis, local treatment for liver metastasis proved to be an independent prognostic factor for overall survival (Table 5).

**Table 4:** Univariate and Multivariate Analyses for Pelvic Failure-Free Survival

Prognostic Factor	Group	PFFS (%)	Univariate	Multivariate	RR	95% CI
Age (years)	< 57	73	0.531	−		
	≥ 57	77				
Duke stage	A or B	88	0.399	−		
	C	64				
Rectal lateral margin	negative	73	0.045	0.05	2.6	1.0-6.6
	positive	51				
Initial CEA (ng/ml)	< 100	67	0.694	−		
	≥ 100	73				
Adjuvant pelvic RT	no	65	0.028	0.04	0.3	0.1-0.9
	yes	81				

*Abbreviations: *CEA = carcinoembryonic antigen; PFFS = pelvic failure free survival; RR = relative risk; CI = confidence interval

**Table 5:** Univariate and Multivariate Analyses for Overall Survival

Prognostic Factor	Group	OS (%)	Univariate	Multivariate	RR	95% CI
Age (years)	< 57	63	0.247	−		
	≥ 57	48				
Duke stage	A or B	68	0.297	−		
	C	53				
Rectal lateral margin	negative	59	0.078	0.029	2.2	1.1-4.5
	positive	37				
Initial CEA (ng/ml)	< 100	59	0.081	0.47	1.3	0.6-2.7
	≥ 100	33				
Liver resection	no	27	0.000	0.0	2.3	0.2-0.6
	yes	76				
Adjuvant pelvic RT	no	49	0.224	-		
	yes	70				

*Abbreviations: *CEA = carcinoembryonic antigen; OS = overall survival; RR = relative risk; CI = confidence interval

In order to minimize the influence of metastatic liver disease on the rates of pelvic failure and overall survival, a subgroup analysis was carried out on the fifty-six patients who received primary tumor resection and local treatment for liver metastasis before receiving pelvic RT. The two year pelvic failure-free survival rates were 64.9% and 82.9% (*p *= 0.05) and two-year overall survival rates were 74.1% and 80.0% (*p *= 0.616) for group S (n = 36) and group S+R (n = 20), respectively.

## Discussion

Metastatic spread of colorectal cancer occurs mainly via the portal system and the incidence of isolated liver metastasis in rectal cancer is seven times higher than isolated liver metastasis in other cancers [2]. The larger volume of blood supply to the liver in comparison with other organs and the tendency of cancer cells to deposit and multiply in the liver after passing through the portal system may explain the organ-specific metastasis of colorectal cancer [7]. Patients with liver metastasis, however, should not be deprived of available treatment options. Finlay *et al*., estimated the mean doubling time of liver metastases from rectal cancer to be 155 days [13]. The relatively slow growth of liver metastasis suggests a potential survival benefit with aggressive treatments in rectal cancer patients who have liver metastasis.

Although rectal cancer patients with synchronous liver metastasis have been treated conservatively with palliative colostomy or bypass surgery in the past, an increasing number of these patients undergo resection of the primary rectal cancer as well as local treatment for liver metastasis. The survival rate of these patients has been gradually improving as an increasing number of patients are undergoing surgical treatments. Improvement in surgical techniques, especially for liver resection, is also a contributing factor for increased survival. However, only 20% of liver metastases are found to be resectable at diagnosis; only 25 to 40% of patients undergoing resection experience long term survival [14-16] since many of these patients die of metastasis to the lungs, bone, and other extra-hepatic sites.

Resection of rectal tumors and treatment of liver metastases can benefit patients by reducing tumor burden and slowing the progression of liver metastasis; thus, these patients are more likely to have a longer disease-free survival and a longer stabilized disease state. However, surgical resection of the primary tumor and treatment of liver metastasis do not eliminate the risk of pelvic failure. Rectal cancer patients with liver metastasis are more likely to have higher T and N stages (i.e., a higher rate of transmural penetration and nodal involvement) [4]. These patients are at a high risk of pelvic recurrence following initial treatment, and thus suffer from shortening of disease-specific survival.

Although postoperative pelvic irradiation is known to increase survival by reducing pelvic failure rates and distant metastasis in locally advanced rectal cancer patients [17], its role is unclear in patients with synchronous liver metastasis. To date, few reports have been published on the benefit of pelvic RT when patients have synchronous distant metastasis. Crane *et al.*, have reported that pelvic irradiation has a palliative role in treating rectal cancer liver metastasis [18]. Eighty patients with synchronous distant metastases from rectal cancer were treated with chemoradiation or chemoradiation followed by primary tumor resection. Symptoms from the primary tumor resolved in 90% of all patients and symptomatic pelvic control rates were 81% and 91% in the chemoradiation and chemoradiation + surgery groups, respectively. Durable pelvic control was safely achieved without colostomy in most rectal cancer patients with synchronous systemic metastases by means of pelvic chemoradiation.

In our study, postoperative pelvic RT reduced the pelvic failure rate in rectal cancer patients with synchronous liver metastasis compared with the patients who underwent surgery alone. Positive lateral resection margin of the primary tumor and adjuvant pelvic RT influenced pelvic failure-free survival as independent prognostic factors. Among the twenty-seven patients who received postoperative pelvic RT, pelvic failure occurred in only two in fourteen patients with early stage liver metastasis (stage I/II) and in two in thirteen patients with late stage liver metastasis (stage III). These results suggest that pelvic RT may benefit all patients regardless of the status of their liver metastasis. However, the number of patients who received resection of the primary tumor despite stage III liver metastasis is small and the role of postoperative pelvic RT in extensive liver metastasis needs to be verified by future studies. In a subgroup analysis consisting of 56 patients who received local treatment for liver metastasis, postoperative pelvic RT also showed improvement in pelvic control rate. Overall survival, however, demonstrated no significant difference between group S and group S+R. Local treatment for liver metastasis was the only independent prognostic factor for overall survival. A prospective study with a larger group of patients and a more thorough follow up may reveal translation of improved local control into survival benefit.

## Conclusion

In the current practice, the majority of physicians do not consider pelvic irradiation as a part of standard treatment when patients are diagnosed with rectal cancer and synchronous liver metastasis. Our study showed that postoperative pelvic RT in rectal cancer patients with synchronous liver metastasis increased PFFS whether or not the patient received a local treatment for liver metastasis. We suggest that these patients may benefit from postoperative adjuvant pelvic RT through an increased pelvic control rate and improved quality of life.

## Competing interests

The authors declare that they have no competing interests.

## Authors' contributions

JK contributed in data collection, performed statistical analysis and drafted the manuscript. YK contributed in design and coordination of the study and critical revision of the manuscript. KK conceived of the study, contributed in patient accrual, participated in study design, and gave final approval of the manuscript to be published. NK, BM, SS, JA, WK, and JS contributed in patient accrual. All authors read and approved the final manuscript.
